# Drosophila Dendritic Arborisation Neurons: Fantastic Actin Dynamics and Where to Find Them

**DOI:** 10.3390/cells10102777

**Published:** 2021-10-16

**Authors:** Lukas Kilo, Tomke Stürner, Gaia Tavosanis, Anna B. Ziegler

**Affiliations:** 1 Dendrite Differentiation, German Center for Neurodegenerative Diseases, 53115 Bonn, Germany; lukas.kilo@dzne.de (L.K.); gaia.tavosanis@dzne.de (G.T.); 2 Department of Zoology, University of Cambridge, Cambridge CB2 1TN, UK; ts587@cam.ac.uk; 3 LIMES-Institute, University of Bonn, 53115 Bonn, Germany; 4 Institute of Neuro- and Behavioral Biology, University of Münster, 48149 Münster, Germany

**Keywords:** neuronal dendrites, dendrite arborization (da) neurons, actin, time-lapse imaging

## Abstract

Neuronal dendrites receive, integrate, and process numerous inputs and therefore serve as the neuron’s “antennae”. Dendrites display extreme morphological diversity across different neuronal classes to match the neuron’s specific functional requirements. Understanding how this structural diversity is specified is therefore important for shedding light on information processing in the healthy and diseased nervous system. Popular models for in vivo studies of dendrite differentiation are the four classes of dendritic arborization (c1da–c4da) neurons of *Drosophila* larvae with their class-specific dendritic morphologies. Using da neurons, a combination of live-cell imaging and computational approaches have delivered information on the distinct phases and the time course of dendrite development from embryonic stages to the fully developed dendritic tree. With these data, we can start approaching the basic logic behind differential dendrite development. A major role in the definition of neuron-type specific morphologies is played by dynamic actin-rich processes and the regulation of their properties. This review presents the differences in the growth programs leading to morphologically different dendritic trees, with a focus on the key role of actin modulatory proteins. In addition, we summarize requirements and technological progress towards the visualization and manipulation of such actin regulators in vivo.

## 1. Introduction

An integral part of most neurons are the dendrites, the subcellular compartment receiving synaptic and sensory input. Dendrites often form a branched arbor, which plays a major role in determining the function and properties of a neuron, as it houses its main input sites [1,2]. This structure is further defined by its shape, length, and complexity, resulting in specific morphologies for each neuronal class, and is tailored to the specific functions and connections maintained by the neuron [3]. Most dendritic trees cover an area so large that they make up the vast majority of the surface of a given neuron. The morphology of dendritic trees defines not only the amount of afferent inputs interacting with the neuron but also subsequent signal propagation [4]. The individual dendritic morphologies of neurons are often highly stereotyped and many of these neurons have been studied extensively over the years to gain a better understanding of the parameters governing dendritic patterning. Dendritic arborization has been studied across different species and in several neuronal types such as the PVD neurons of *Caenorhabditis elegans* [5,6], or the Purkinje cells of rodents [7] and multiple neuronal types of *Drosophila melanogaster*, including dendritic arborization (da) neurons [8], lobula plate tangential cells [9], and motor neurons [10]. Each of these model systems produces complex yet distinct dendritic arbors. Among those highly morphologically stereotyped neurons are the da neurons of *Drosophila melanogaster* larvae [8]. These sensory neurons can be subdivided into four classes with distinct dendritic arborization patterns. In particular, the rather simple class one neurons (c1da) and the highly branched class four neurons (c4da) have been extensively studied since their first characterization [8]. Da neurons can be easily visualized in vivo and can be imaged without any major damage to the animals. Furthermore, they can be easily manipulated in a noninvasive way, due to the large genetic toolbox inherent to the *Drosophila* model system. To achieve these highly stereotyped dendritic trees, their differentiation needs to be rigorously regulated. The mechanisms governing the outgrowth of the primary dendrites, their branching, and the stabilization of newly developed branches, all need to work coherently to achieve a fully functional dendritic tree. In recent years, with the continued support of modeling approaches, growth programs underlying the patterns of dendritic arborization and organization have been identified [11,12,13,14]. These models suggest deterministic and stochastic steps in a segmented/iterative growth program. Very recent studies even propose similar generalized growth programs for different neuron types [11,12,13,15]. Implementation of these programs is mediated by transcription factors [16,17,18,19] and other modulating stimuli such as external interaction cues [20,21,22]. Ultimately the changes elicited by these stimuli need to be converted into changes to the neuronal cytoskeleton that define the morphological characteristics of the dendrites [21,23].

Actin, the most dynamic part of the cytoskeleton plays a major role across all aspects of dendritic arborization [24]. Actin is the driving force in the outgrowth of a dendritic tip [25]. The branching of dendrites is often predated by the accumulation of F-actin, with actin patches playing a key role in dendritic branching itself [26,27]. Furthermore, dendritic branch stability is increased by actin crosslinking or the introduction of microtubules (MTs) as an additional cytoskeletal scaffold [28,29]. Many recent technological advances enable high-resolution and high-speed in vivo examination of dendritic growth, whilst performing acute and spatiotemporally confined manipulations [27]. Studies with this degree of resolution are a viable and necessary step to deepen our understanding of actin-related dendrite dynamics. Here, we aim to give a comprehensive overview of the techniques enabling these studies and insights gained employing such techniques on the intricate dynamics of actin in the growing dendrite.

## 2. The da Neurons of the *Drosophila* Larva

The da neurons of the peripheral nervous system (PNS) of *Drosophila* larvae are embedded between the body wall muscles and the transparent cuticle, spreading their arbors in an almost 2D manner [8]. Three to five neurons per da class are arrayed along the dorso-ventral axis of each abdominal hemisegment [8]. Their distinct neuronal identities become defined during development by combinations of transcription factors [30,31,32,33,34].

**Figure 1:**
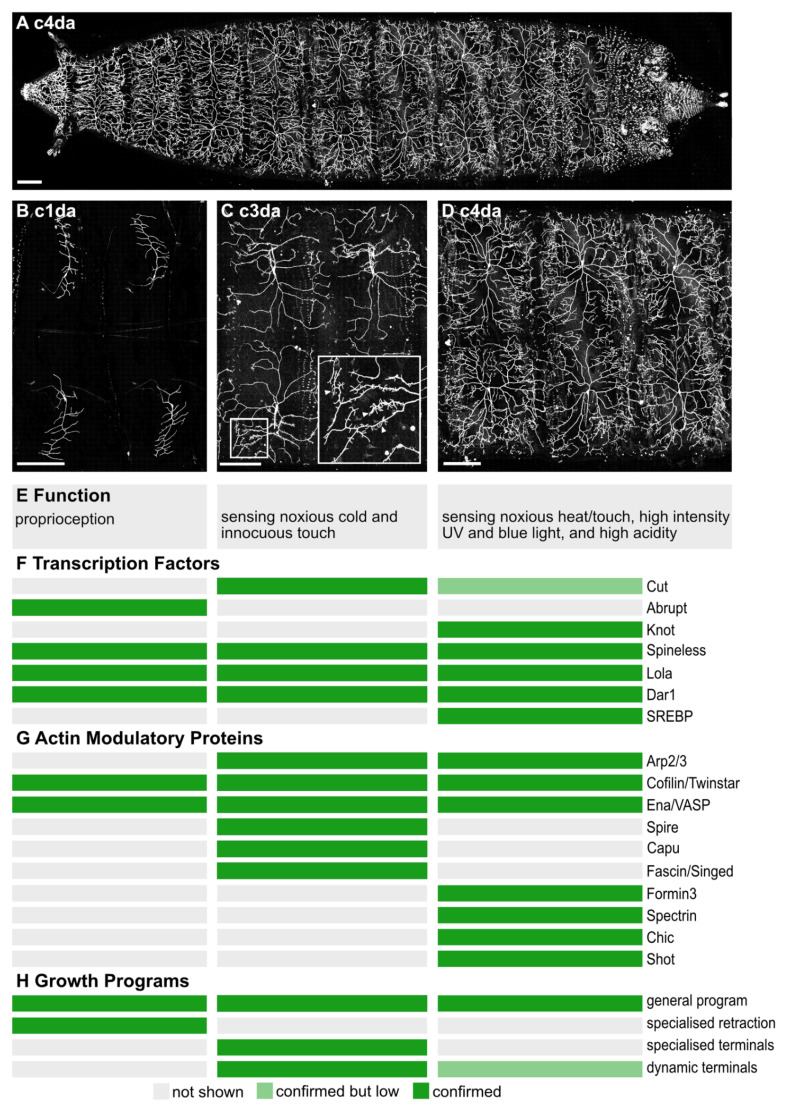
Structure and function of da neurons. (**A**–**D**) Living wandering third instar larvae were immobilized in between a glass slide and a coverslip. C1da (**B**) and c3da neurons (**C**) were specifically labeled using *Gal4^221^* or *Gal4^19−1^* driven expression of *UAS-CD4::GFP*. C4da neurons (**A,D**) were marked by *pickpocket*-driven *CD4::GFP* expression (scale bars = 200 µm). Confirmed presence of (**F**) transcription factors linked to actin, (**G**) actin modulatory proteins, or (**H**) growth programs in the c1da (**left** panel), c3da (**middle** panel), or c4da (**right** panel) neurons are shown with green bars. When the relative levels of expression or the dynamics were low when comparing between neuron classes this was conveyed by a light green bar. References in the main text.

The three c1da neurons are proprioceptive and respond to the peristaltic contractions or bending of the larval body wall [35,36]. Ventral c1da neurons display main branches aligned with the dorsal–ventral axis of the hemisegment and produce few straight lateral branches that point either towards the posterior or the anterior hemisegment boundary [8] (Figure 1B). These dendrites thus display a characteristic comb-like shape. The lateral dendrites of c1da neurons run along the direction of contraction and are sequentially deformed within the consecutive segments during crawling. This branch orientation is thought to maximize membrane curvature during larval crawling and thereby tightly couples c1da dendrite morphology to the neurons’ proprioceptive function [12,37]. The four c2da neurons also exhibit a simple dendritic morphology, but with longer branches than c1da neurons. Functionally, they are the least characterized and so far no unique functional feature was assigned to that class [8,38]. They contribute to the perception of innocuous touch and their optogenetic activation leads to a stereotyped defensive rolling [38,39]. The five c3da neurons (Figure 1C) respond to noxious cold and innocuous touch [8,39,40]. Their morphology is characterized by long main branches decorated with actin-enriched short, straight branchlets (Figure 1C, inlet arrowheads). Their optogenetic activation leads to a contraction of the animal [38]. The most complex dendritic morphology is achieved by the space-filling c4da neurons (Figure 1D) that, with their highly branched dendrites, cover each hemi-segment and thus the whole animal (Figure 1A) [8]. Dynamic growth and retraction of high order branchlets decline over time but are still detectable at the latest larval stages [27]. These highly polymodal neurons express a set of receptors that enable them to respond to noxious heat, noxious touch, high intensity of UV or blue light, and high acidity. They represent the primary nociceptors of the *Drosophila* larva and their excitation, whether by mechanic or ectopic optogenetic stimulation, evokes a characteristic rolling behavior immediately followed by increased locomotion speed [38,41,42,43,44,45,46,47]. C4da dendritic complexity can be altered by environmental factors (such as food conditions [48,49]) as well as through genetic manipulation. Here multiple studies have reported a link between the complexity of c4da neuronal dendrites and their sensitivity to noxious environmental stimuli which again highlighted the importance of a correctly established dendritic morphology for the function of these neurons (Figure 1E) [19,50].

The highly stereotyped dendritic fields of the different da neuron classes are shaped by early transcription nodes (Figure 1F). Shortly after the initial establishment of da neurons as a model system of choice, the homeodomain homolog transcription factor Cut was found to influence branching. Loss of Cut reduces higher-order branches in both c3da and c4da neurons. For c3da neurons Cut is especially important as it defines their specific, short terminal branch heavy morphology [8,31]. This was later followed up on by Corty et al. who discovered the role of Cut on a repressor cascade inhibiting higher-order branching, effectively derepressing downstream branching factors [17]. Other well-investigated transcription factors determining dendritic and thus functional identity are the zinc finger transcription factor Abrupt and the Collier/Olf1/EBF (COE) protein Knot. Both these transcription factors influence the general dendritic shape of c1da (Abrupt) or c4da (Knot) neurons. Knot induces the expression of the MT severing protein Spastin, and thereby promotes the outgrowth of *bona fide* dendrites [32]. Spastin expression is conversely inhibited by the overexpression of the transcription factor Dendritic arborization reduced 1 (Dar1) which results in a larger dendritic tree and more stable MTs in terminal branches [34]. Knot simultaneously also promotes the bifurcation of lower-order branches by regulating the atypical myosin Myo6 [29]. Many da neurons undergo metamorphosis during the pupal phase. Shortly after pupation they prune away their dendrites and regrow a fully new dendritic tree. Yoong and colleagues have studied dendrite regrowth during pupal stages in c1da neurons. With high-resolution time-lapse imaging, they revealed that Myo6 is responsible for introducing anterograde-directed MTs along extended actin filaments into the dividing dendritic tip of pupal c4da neurons, thereby reducing actin dynamics and stabilizing the dendrite. Lastly, Abrupt plays a major role in determining the comb-like shape of the larval c1da neurons, even evoking similar arrangements of the dendritic tree in the other da neurons if expressed ectopically [33,51]. The target genes downstream of transcription factor activity are plentiful and often function in a dosage-dependent manner [16,52]. Although several of the immediate effectors of these transcriptional nodes could be identified most of the direct programs leading to cytoskeletal adjustments are still unknown (Figure 1G). Recently, a transcription factor not conventionally associated with cytoskeletal dynamics could be found to influence c4da neuronal morphology. Specifically, sterol regulatory element-binding protein (SREBP), which regulates lipid synthesis rates, is required for branch elongation in c4da neurons. In mutant animals, c4da neurons develop short terminal dendrites morphologically similar to the small terminal branches of c3da neurons. These morphological changes in dendrite morphology went along with a hypersensitive reaction of the mutant animals towards noxious stimuli indicated by more frequent and multiple rolling [19].

## 3. Dendrite Differentiation: Noninvasive Long Term In vivo Imaging Identifies Distinct Phases

Before studying how the absence of specific actin modulatory proteins would affect dendrite differentiation, it is essential to understand the logic of how neurons are developing in their natural environment. The expression of fluorescently-tagged proteins to visualize subsets of da neurons becomes detectable at the embryonic stage E16 [31]. At that stage, da neurons are already polarized and extend their long main dendrites. A recent study investigated the embryonic stages of c1da neuron dendrite differentiation and revealed that the lateral branchlets of c1da neurons underwent repeated cycles of extensive dendritic branch formation and retraction [12]. This process temporarily gave rise to a much higher total number of lateral dendrites, as compared to the final c1da dendritic tree of a third instar larva, and led to the outgrowth of higher-order branches, which in the case of c1da neurons, are never found in the fully developed dendritic tree. The maximum branch number of c1da neuronal dendrite was achieved at embryonic stage E18.5-19. Hereafter, c1da neuronal dendrites entered the so-called retraction phase that reorganizes the dendritic tree structure. In that phase, those branches that had grown from the main branches with a flat angle were preferentially retracted while lateral branches that had branched from the main branch with a high angle preferentially became stabilized leading to a dendritic tree with almost parallel lateral branches. A mechanism underlying this pattern involves homotypic repulsion mediated by the cell surface molecule Down syndrome cell adhesion molecule 1 (Dscam1) [15]. During the retraction phase, no new branches were added and by the end of the embryonic life dendritic trees of c1da neurons have almost reached their final shape. The final step of dendrite differentiation of c1da neurons, the stretching phase, occurs during larval life [12]. Here, the already preformed dendritic arbor needs to scale with the growth of the animal. This phase is marked by a rapid increase in total dendritic length and total dendrite spanning area while only a few new branches were added [53]. A complementary study concentrated on the developmental maturation of dendrites focusing on the space filling and highly complex c4da neurons [54]. C4da neuronal dendrites are characterized by their dense network of non-overlapping dendrites that cover up the whole surface of the larval body wall. Despite their complexity, c4da neurons are optimally built to constrain total dendritic length [54]. They do so by following the branching principles of minimum spanning trees, which uses the minimal total length required to fill an area with a predefined dendritic density [11,54]. In contrast to c1da neurons, in which almost no new branches are added during the larval growth phase, c4da neurons gain newly formed dendritic branches during larval life, which allows these neurons to maintain their overall space-filling characteristics. However, as for the c1da neurons, the basic morphological characteristics of the c4da neuronal branches are conserved during the larval growth period and their main branches extend by a stretch-and-fill process [54]. Detailed data of early differentiation are not available to our knowledge for c3da neurons. Nonetheless, it has been recently shown that their main branches also follow the requirements of a minimum spanning tree. However, the properties of their characteristic short terminal branchlets cannot be reproduced by such models. Instead, a second step is required to distribute short dendritic branches along the main branches with increasing probability towards the terminals [13].

Taken together, the c1da, c3da, and the c4da neurons remarkably share the dendritic growth parameters of their main branches, which extend through a stretch-and-fill mechanism [54]. However, their terminal or higher-order branches are neuron-type specific and thus must have specialized growth parameters. The distinct morphological structures that define a neuronal cell type must ultimately have distinct cellular properties, defined by the cytoskeleton. Therefore, many studies on dendrite differentiation have focused on the identification of regulatory proteins controlling the organization of the cytoskeleton, that are involved in general dendrite growth mechanisms as well as in shaping specific dendritic structures.

## 4. Tools to Visualize and Manipulate Actin Dynamics

Technological advances over the past two decades have improved the means to visualize dendritic actin dynamics in vivo quite significantly [55,56]. Non-disruptive tagging of actin as well as live imaging techniques have become more sophisticated. Actin nucleators and other proteins, directly interacting with actin, can be traced by direct tagging with fluorescent proteins. In addition to the means of visualization [36], methods of targeted interference and/or the initiation of actin-related biochemical processes [57] have noticeably improved [58,59]. Some of these methods will be further discussed in this section.

**Figure 2:**
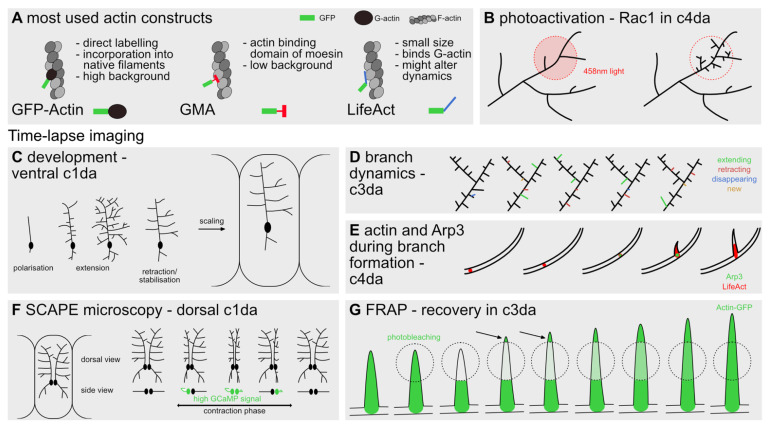
Tools for in vivo imaging and manipulation. (**A**) The three most commonly used constructs for visualizing actin in vivo. Schemes show how GFP-actin, GMA and LifeAct are incorporated into an actin filament. (**B**) Example of how a photoactivatable construct can help understanding the functional role of a specific molecule or cascade in dendrites. PA Rac1 activation in c4da neurons with blue light (458 nm) leads to the formation of small dendritic branchlets [27]. (**C**–**G**) Examples of how time-lapse imaging was used to understand different stages of dendrite branch formation. (**C**) Long-term imaging of the c1da neurons throughout embryonic and larval development, elucidating the sequence of differentiation stages [12,15]. (**D**) High-resolution imaging in the terminal branches of c3da neurons to analyze their constant dynamics. Analysis of branches newly forming, extending, retracting, and disappearing can be monitored within a range of 30 min [13]. (**E**) Dynamics of actin patches (LifeAct, here shown in red) along dendritic branches of c4da neurons, accumulating before new branch formation and extending into the newly formed branchlet [26]. Labeled Arp3 (shown as a green dot) accumulates transiently at the site before a new branch is formed and disappears shortly after branch formation [27]. (**F**) High-speed imaging of c1da neurons with SCAPE microscopy allows monitoring the deformation and the intracellular calcium activity of the dendrites in freely moving larva [36]. (**G**) Bleaching and subsequent recovery of the GFP-actin signal at the tip of a growing dendritic branchlet of a c3da neuron. The most commonly used bulk kinetics approach method to study actin structure was performed in vivo to define the orientation of actin filaments in the extending branchlet [13].

Direct visualization of F-actin dynamics in vivo in *Drosophila* has been mainly made possible by three different tools, the GFP-tagged actin-binding domain of Moesin (GMA), LifeAct::GFP, and GFP-actin (Figure 2A). GMA is a fusion protein of the actin-binding domain of *Drosophila* Moesin with a GFP tag substituting the functional moesin/ezrin/radixin (MER) domain. This fusion protein does not interfere with the developing cytoskeleton and distinctly labels F-actin during nucleation, without unspecific background fluorescence [60,61].

Another probe used to visualize F-actin is LifeAct, a 17 amino acid long peptide derived from budding yeast. This short actin-binding sequence can be conjugated with regular fluorophores such as GFP to directly label F-actin. For this construct, similar to GMA, no interference with the physiological properties of the tagged actin could be found in the initial study [62]. However, later studies found detrimental effects for high LifeAct concentrations in *Drosophila* oogenesis [63], which could recently be related back to a competition of LifeAct with binding sites for Cofilin and Myosin [64]. Nonetheless, a recent study compared the effects of both LifeAct::GFP and GMA for expression in c4da neurons and could not find significant differences for commonly used dendritic tree statistics, even in vivo [26]. Aside from these dynamic probes more classical ones, such as GFP-actin or phalloidin, have been used. GFP-actin is an ectopically-tagged version of G-actin which is prone to high background fluorescence [65], while phalloidin can only be used in fixed preparations [55] (Figure 2A).

To directly probe actin dynamics in vivo, spatiotemporally confined manipulation of the putatively involved factors is necessary. For such acute manipulations, optogenetics and temperature-sensitive mutants are best suited. Here, the *Drosophila* larval da neurons are again advantageous, since they are situated so close to the body wall, that temperature shifts quickly reach the desired neuron. Similarly, the transparent epidermis removes many obstacles usually associated with the activation of optogenetic tools.

For example, the small GTPase Rac1 has been adapted as a photoactivatable version [57]. In this case, a light-sensitive domain obstructs a constitutively active mutant of Rac1 (PA Rac1) until a light-induced conformational change mobilizes the obstructing domain (Figure 2B). This tool has successfully been used to visualize the interaction of Rac1 and Arp2/3 in c4da neurons [27]. Other applications of optogenetic methods include caging and uncaging of cellular substrates, clustering of proteins, or their removal to specific subcellular domains [58]. The main hurdle when applying such attractive optogenetics approaches is the fact that for most proteins no tools are readily available, and they subsequently have to be custom made for the specific scientific question.

These many different methods of tracing or manipulating actin dynamics in vivo need suitable means of visualization. Depending on the type of dendrite analyzed the correct means of visualization has to be chosen to provide the appropriate spatiotemporal resolution. For da neurons, depending on the chosen neuron different phases of the overall dendritic growth program can be examined. During embryonic development, the differentiation steps leading to the highly stereotyped morphology of c1da neurons can be followed easily by live imaging [12] (Figure 2C). When focusing more on overall dendritic branch dynamics, c3da neurons and their small terminal branches developing in the later larval stages are appropriate to further examine branching, extension, retraction, or stabilization of individual branchlets [13] (Figure 2D). Lastly, the highly dynamic nature of the c4da neuron branches is well suited for discrete manipulation of actin-binding proteins via optogenetics, as described above [26,27] (Figure 2B,E).

The above-mentioned time-lapse series can be performed using conventional confocal or 2Photon (2P) microscopy with immobilized samples. Specifically, time-lapse imaging of the da neurons, of only partially restrained *Drosophila* larvae, has been recently achieved by multispectral, high-speed, volumetric swept confocally aligned planar excitation (SCAPE) microscopy [36], thereby allowing to visualize physiological excitation patterns of the neurons. A map of physiological responses during larval maturation could be very instructive for determining activity-dependent dendritic growth and the underlying stimuli for actin cytoskeletal regulation (Figure 2F).

To visualize the motility of actin in vivo specifically fluorescence recovery after photobleaching (FRAP) can be employed. This method uses a strong laser to bleach a fluorophore; the subsequent fluorescence recovery in live cells allows a description of the time-course and pattern of motility of a tagged protein, e.g., actin. Specifically, the directional recovery of a fluorescent signal indicates the bulk orientation of actin filaments. Figure 2G shows how FRAP was used to describe the dynamic properties of actin in a growing dendrite branchlet [23,27].

Lastly, visualization of actin dynamics in vivo, particularly in da neurons, profits heavily from conditional expression systems and other genetic tools available in the *Drosophila* model system. Utilizing these tools, among many other advances, a broad categorization of growth patterns across da neurons became possible, the results of which will be discussed further in the following sections. 

## 5. Dendrite Branching

The effects of actin modulatory proteins on dendritic arborization have been studied in different model systems, including the *Drosophila* central nervous system. For example, in lobula plate tangential cells of the *Drosophila* visual system it could be shown that phosphorylation of Moesin promotes actin-dependent dendritic arborization [66]. Nonetheless, to ease comparability of the many actin modulator proteins discussed below, this review focusses on observations made in the powerful model system of the larval *Drosophila* da neurons.

Recent work using live confocal imaging of MTs, labeled by *UAS-mCherry::Jupiter* and simultaneous labeling of F-actin via GMA::GFP have proposed a computational simulation of arbor structure in these neurons. The model, which holds true for the c1da and c4da demonstrated that dendrite arbor length is highly correlated with the MT quantity while dendrite branching is highly associated with F-actin. More MTs led to a greater arbor length and branches tended to terminate once MT density got lower. At the same time bifurcations were strongly correlated with local F-actin enrichment [67]. The formation of filopodia-like branchlets has been widely studied in c3da and c4da neurons. The site of de novo branch formation could be predicted by the accumulation of tagged GMA or LifeAct [26,27]. Additionally, the presence of polymerized F-actin at the base of dendritic branches could be shown by electron microscopy [27].

However, which are the molecules upstream of F-actin accumulation at sites of branch formation? Which proteins are directly involved in organizing F-actin at these sites in the morphologically different neurons? And how is the activity of these proteins regulated?

An in vivo analysis in differentiating c4da neurons revealed that the enrichment of genetically encoded GMA and LifeAct appears at the base of dendritic branch points shortly prior to branch formation in about 90% of all analyzed de novo branch formation events [26,27]. These actin patches were more frequently found in terminal dendrites which also undergo more frequent extension and retraction than non-terminal ones. Additionally, actin patches were more frequently found in c4da neurons of early third instar larvae, in which the dendritic tree morphology is more dynamic than in the older mid-third instar larvae. Actin patches were shown to move rather slowly in anterograde and retrograde direction with a speed of approximatively 1 µm/min and mark a preferential site of a future dendrite branching. For example, in c4da neurons actin patches emerged on average for 1 m and 54 s before a new branch was formed. These results are in agreement with previously published literature which suggests that high actin dynamics correlate with dendrite branching events [23]. The appearance of an actin patch led to branch formation in only 24% of the analyzed cases. However, the frequency of a branch forming was increasing if the actin patch got stalled [26].

F-actin assembly and disassembly are necessary to maintain the dynamics that leads to branch formation. This has been partially shown by the expression of mutant actin^G15S^, which is incorporated together with WT actin into F-actin filaments, increasing F-actin stability, most likely by reducing Cofilin binding probability. Actin^G15S^ expression and thereby decreased actin dynamics reduced actin patch formation by about 50% and led to a loss of dendritic complexity in c4da neurons [26]. The dynamic nature of actin patches is in part regulated by Twinstar (Tsr), the *Drosophila* homolog of Cofilin with a prevalent F-actin severing function [13]. Loss of Tsr led to fewer, more static, and smaller actin patches and, in turn, to a reduction in newly formed branches in *tsr*mutant c4da neurons. It is therefore believed that the actin-severing function of Tsr produces short actin filaments which could subsequently elongate in different directions, a necessity for dendrite branching [13,26]. Surprisingly, only 35% of newly formed dendritic branchlets in c3da neurons were pre-marked by actin patches suggesting that morphologically different neurons employ different strategies for F-actin enrichment at dendrite branching sites [26]. However, terminal branch formation in c3da neurons seems also to greatly rely on Tsr activity as GFP::Tsr could be localized to small terminal branchlets and loss of Tsr activity led to a huge loss of small terminal branches in these neurons [13,68].

Actin polymerization at terminal branch formation sites is regulated by different kinases [23,69,70]. In c3da neurons, small terminal branch formation seems to rely predominantly on the activity of Calcium/calmodulin-dependent protein kinase II (CaMKII). Studies mostly conducted on cultured hippocampal neurons suggested that CaMKII autophosphorylates after Ca2+ influx which in turn activates a cascade leading to *de novo* filopodia formation (reviewed by [71]). Specifically, in the c3da neurons, time-lapse imaging revealed that the overexpression of a constitutively active form of *Drosophila* CaMKII^T287D^ increased the amount of newly formed branches and thereby increased the number of small terminal branches [23].

Negative regulators of neuronal branching behavior are the Tricornened Kinase (Trc) and its physical interaction partner Furry (Fry) [69,72]. Loss of both proteins results in heavy overbranching in all types of da neurons [69]. Trc physically interacts with Sin1, an essential component of the Target of Rapamycin Complex 2 (TORC2), which is a known regulator of cytoskeleton organization [73]. *Sin1* and *rictor* mutant c4da neurons phenocopied the overbranching phenotype of *trc* and *fry*. Both phenotypes could be rescued by the expression of a dominant active form of Trc proving the interaction between Trc/Fry and the TORC2 complex in the context of dendrite branching [72]. Thousand and one (TAO) kinase is an additional negative regulator of dendrite arborization in c1da and c4da neurons. Quantifying the number of added dendritic termini of *tao* mutant neurons throughout larval development revealed that even in c1da neurons, which normally grow by just extending the embryonically formed dendrites, new termini would be constantly added until the end of the larval life. A more precise analysis of dendrite extension and retraction speed has shown that Tao does not act on dendritic branch de novo formation but rather reduces extension/retraction kinetics of dendritic branchlets. A quantification of actin levels using LifeAct::GFP revealed that knockdown of *tao* via RNAi increased F-actin levels in the main branches and in terminal branches indicating that Tao acts upstream of F-actin accumulation and distribution [70].

Rac signaling is a well-established regulator of dendritic growth and remodeling (reviewed by [74]). In da neurons, the Rac family member Rac1 seems to play an essential role in dendrite morphology and lays downstream of CaMKII and Trc/Fry signaling. Absence of Rac1 decreases the dendrite branching of c4da neurons. On the other hand, Rac1 overexpression induces small terminal branch formation and does so even in neurons, such as c1da neurons, that normally lack small terminal branches [23,75]. Rac1 activity may be regulated by different upstream factors. Co-immunoprecipitation of Trc with Rac1 indicated a direct interaction of those proteins. Furthermore, the expression of dominant-negative Rac (RacN17) could restore the dendrite overbranching phenotype in Trc^DN^ expressing c4da neurons [69]. Whether CamKII also directly or indirectly acts on Rac1 activity in da neurons remains unknown.

Downstream of these kinases and Rac1 are the Wiskott–Aldrich syndrome protein (WASP) and WASP family verprolin-homologous protein (WAVE) complex. WASP family members can directly bind to the plasma membrane via their basic domain and recruit actin nucleators such as the actin-related protein 2/3 (Arp2/3) complex to the site of nucleation via their acidic and their connector domain [76,77]. The *Drosophila* genome encodes multiple WASP family members (WAVE/SCAR, WASP, and WASH), however, only the knockdown of *wave* in c4da neurons phenocopied the loss of *rac1* mutants [27]. Consistent with its suggested role within this pathway, *wave* null mutant (*scar^Δ37^*) c4da neurons have a simpler dendritic arbor at the end of the larval life. This was explained by a reduced number of newly formed branches [27]. WAVE is the primary regulator of Arp2/3 complex-dependent morphological events in the fruit fly [78] and the expression of a mutant form of WAVE (*wave^ΔVCAmyr^*) which lacks the Arp2/3 interaction domain reproduced the *wave* null mutant phenotype [27]. The Arp2/3 complex consists of multiple subunits including Arp2, Arp3, and Arpc1 which are encoded by distinct genes [79]. RNAi-mediated knock-down of homozygous null mutant cell clones for the different Arp2/3 complex members displayed strongly reduced total branch numbers of c3da and c4da neurons while main dendritic branches remained unaffected [27]. Previous studies already suggested that actin patches accumulating at sites of dendritic spine formation contain Arp2/3 [80]. The study provided in vivo data supporting the notion that dendrite branchlets also require Arp2/3 by imaging a transient accumulation of GFP-tagged Arp3 (Arp3::GFP) on average 60 s prior to de novo branch formation in c4da neurons [27]. After the formation of the branchlet, Arp3::GFP signals at the base of the newly formed branch were gradually decreasing.

Taken together, the da neurons served as a *bona fide* model to investigate the importance of properly regulated actin dynamics during dendrite branching events. Here it became evident that two major components are necessary for dendrite branching. At first, a new branching site is marked by an actin patch and Arp2/3 accumulation and the branchlet starts elongating. However, whether this branch remains and becomes stabilized seems to depend on the kinetics that regulates the extension/retraction rate of such branchlets. 

## 6. Dendrite Extension and Stabilization

A nascent branch undergoes maturation before it becomes a lasting dendrite. Initial dendritic branches need to elongate, reach a substrate or synaptic partner with which to interact, and form lasting connections. More often than not an exploratory branch cannot fulfill these conditions or it intrudes upon territory already occupied/covered by its sister branches, leading to the retraction of the branch. Many molecules and pathways which play a role in the stabilization or destabilizing of actin scaffolding have been already identified.

In c3da/c4da neurons, the *Drosophila* homolog of Fascin, called Singed, was found to play a role in the elongation of newly branched, actin-rich dendrites. Singed tightly cross-links actin into bundles [81]. Singed interacts genetically with the transcription factor Cut, which is highly expressed in c3da neurons. Ectopic expression of Cut results in additional c3da-like spiked protrusions in other classes of da neurons. This effect did not occur in a *singed^36a^* loss of function mutant background, suggesting that Singed is a downstream effector of Cut function. Co-localization of GFP::Singed to these protrusions in extending c3da neurons reinforces its role in dendrite extension, while dendrites lacking Singed are more likely to retract [82]. The effects mediated by Singed are modulated by its phosphorylation, as shown by the expression of a phosphomimetic mutant version (*sn^S52D^*). Thus, phosphorylated Singed/Fascin is less likely to locate towards newly formed branches and promote maintenance/elongation of these spiked protrusions [82].

One major interaction partner of Singed bundled actin filaments is the actin polymerizing factor Enabled (Ena)/vasodilator-stimulated phosphoprotein (VASP). Ena could be shown to protect the barbed end of F-actin from capping, arranging F-actin into bundles to be cross-linked by Singed, and subsequently coordinating F-actin extension along the growing bundle [83]. These findings in *Drosophila* macrophages could be recently extended to c3da neurons, by examining dendritic trees in specific *ena* mutants (*ena^210^*) over 30 min of dynamic elongation and retraction [13]. Similar to previous reports [84], Ena slowed elongation down but likely increased actin bundling. Due to their known molecular interactions, the cell surface receptor Roundabout (Robo) has been suggested as an upstream regulator of Ena/VASP in this context, as *robo* mutants phenocopy the *ena* mutant neuronal phenotype [84]. Another study confirmed those results with a similar phenotype in *ena* dominant-negative mutants (*ena^46^*) [26].

The actin interacting molecule Spectrin promotes the stabilization of extended branches. Spectrin was found also in dendritic spines and dendrites of cultured hippocampal neurons, organizing F-actin into actin rings, which provide regularly interspersed scaffolding to the cell membrane, thereby promoting dendritic stability [38,85]. Similarly, knock-down of β-III-Spectrin via RNA interference in c4da neurons (*ppk-Gal4*) reduced distal dendrites. Expression of a β-III-Spectrin variant (βSpec^SCA5^) with strongly increased actin affinity prevented the even distribution of Spectrin across the dendritic tree. These results revealed a function of Spectrin in stabilizing branches, by protecting them from retraction. This could be further confirmed by increased distal branch length in *UAS-βspec^SCA5^* overexpressing c4da neurons [86].

Aside from the interaction of Singed and Ena/VASP promoting dendritic elongation, few other proteins interacting with actin play a role in dendritic branch stabilization. Spire, which is an actin nucleator, is important for F-actin-rich terminal branches [27,87]. Spire is directly inhibited by the transcription factor Longitudinals Lacking (Lola), which promotes the expression of the already discussed transcription factors Cut and Knot [87]. As displayed by *lola* RNAi and *lola* single-cell mutant clones generated by MARCM, an increase in abnormal F-actin-rich branches can be found in c4da and c1da when Lola is absent and no longer repressing *spire* expression in these neurons. Previous studies could show a functional interaction of Spire with the *Drosophila* Formin 2 homolog Cappuccino (Capu), in vitro and in vivo, promoting the formation of actin meshes [88,89]. Recently, Capu has also been suggested as a nucleation-promoting factor of Spire and their synergy described in different conditions [88,90]. In c3da neurons, the interaction of Capu and Spire has been proposed to be essential for branch elongation, following initial branching in c3da neurons. This observation was supported by a strong genetic interaction between these two factors. Thus, Spire and Capu might have an actin nucleating role and replace the Arp2/3 function past initial branch formation [13].

Another member of the Formin family, Formin 3, also mediates branch stability. Specifically, Formin 3 increases the likelihood that a branch becomes stabilized. Loss of Formin 3 leads to reduced arborization of *Drosophila* c4da neurons. Here, Formin 3 knockdown not only reduced the amount of actin-rich terminal branches but also led to a significant decrease in stabilized MTs. In contrast, overexpression of Formin 3 in c4da neurons promoted general dendrite thickness, as well as increased distal dendritic branch length significantly. These effects occur alongside a relative increase in MT and F-actin density in proximal parts of the dendritic tree, visualized by co-labeling with mCherry::Jupiter and GMA, respectively. Formin 3 thus plays a major role in dendritic stabilization, by recruiting MTs to F-actin-rich branches [50]. Due to low levels of Formin 3, the affected larvae have lost their sensitivity to noxious heat stimuli. This is similar to the MT guiding and stabilizing effects of Short Stop (Shot), which interacts with actin rings in the axon [91]. *Shot* mutant MARCM clones have been reported to lead to a reduced number of dendrites in c4da neurons but unfortunately, no further analysis was performed [26]. The same study also showed RNAi-mediated knock-down of *chickadee* (*chic*) expression in c4da neurons. Chic is the only *Drosophila* Profilin homolog, a family of proteins that bind actin monomers and provide the major cellular pool of readily polymerizing ATP-actin monomers. *Chic* mutant c4da neurons have a reduced number of dendritic branches and the size of actin patches traveling along dendrites without altering their velocity [26].

The factors promoting elongation of the nascent dendritic branch such as Capu/Spir, Ena/VASP, or Singed/Fascin are closely intermingled with the ones promoting dendritic stability such as Formin 3 and β-III-Spectrin. However, the molecular switch determining whether a branch remains stable is yet unknown. Palavalli and colleagues could show via time-lapse imaging of c1da neurons, that new branches never retract past branching sites. Indicating that one of the prerequisites for branching is a fully stabilized originating branch [15].

## 7. Conclusions and Future Perspectives

Throughout development, intracellular and extracellular signals converge onto the cytoskeleton to arrange and rearrange dendrite morphology to the needs of the neuron. Genetic tools available in *Drosophila* improved optical imaging and combining it with computational analysis is enhancing our ability to understand the single elements regulating dendrite morphology and dynamics. Here, we summarized recent studies that each have looked at subsets of actin modulatory proteins and provide an overview of the morphological and functional outcomes of their interactions, cumulating in overarching concepts for dendritic wiring patterns.

Visualization of actin in vivo in the da sensory neurons of *Drosophila* has enabled the description of different actin structures and dynamics, ranging from patch-like actin accumulations at the base of a newly forming branch to the dynamic assembly of actin at the tip of a growing dendrite [26,27]. Manipulation of actin and its regulators in this in vivo system has provided complete mechanisms, such as the interactions required for dendrite branching through Rac1, WRC, and the Arp2/3 complex, and will continue to push the field towards in vivo analyses of actin modulatory proteins [27].

When studying an actin modulatory protein in a specific neuron, we cannot neglect the importance of the structure–function relationships that govern that given neuron. Loss of function of an actin modulatory protein can lead to different morphological phenotypes in the dendrites of different classes of neurons due to, e.g., different compensatory mechanisms or different levels of competition over a finite pool of G-actin [92]. The three-dimensional structure and dynamics of actin within a dendritic branchlet are regulated by the coordinated action of specific subsets of actin modulatory proteins. In the age of big data science, we are aware that we cannot concentrate on one protein at a time when we are trying to understand an entire three-dimensional dynamic structure. We must find ways to thoroughly analyze, standardize conditions and compare neuron types to fully understand even a simple process, such as the extension of a single dendritic branch [13,93].

We might be close to understanding how the actin cytoskeleton can coordinate dynamic extension and retractions and assemble a dendritic branch with the help of actin modulatory proteins. We are, however, only starting to understand how the assembly of dendritic branches is affected by the activity of the neuron and how a specific morphology is connected to neuronal function and subsequently to the activity patterns observed in the behaving animal. Advances in high-speed 3D imaging allow imaging of the different da neurons in unconstrained larvae in real time [36,37]. Rapid imaging of GCaMP activity of wildtype da neurons and neurons mutant for actin modulatory proteins could finally address these questions in a freely moving animal.

Clarifying how dendrite morphology is established and how it is linked to the function of the neuron requires modelling neurons throughout development and understanding the underlying growth rules [54]. Although influenced by many factors, the growth program of da neurons can be subdivided into a two-step process. Firstly, general dendritic outgrowth and secondly, refinement, shaping the specific morphologies [12,13,15], allowing for a concrete description of developmental phases as well as general mechanisms that might be responsible for the refinement of dendrites. The specialization step is tuned to the specific input the neuron is receiving and is mostly affecting the smaller actin-enriched branches [13]. Comparing actin modulatory proteins in this specialization step between different neurons will show which combinations are responsible for the different morphological traits that we can see in dendritic arbors.

Future work will continue to strive towards a comprehensive understanding of specific spatiotemporal dynamics of actin modulatory proteins. Therefore, it is necessary to unravel their interactions with each other and with the actin cytoskeleton as these actin modulatory proteins represent key factors underlying the generalized dendritic arborization. Unfortunately, in many cases, the endogenous localization of actin modulatory proteins regulating actin dynamics within dendrites has not been elucidated in vivo. Such experiments should clearly be the goal of future studies. However, a recent study provides a comprehensive model of the either confirmed or putative localization of Arp2/3, Spire, Ena, Tsr, Capu, Formin2, and Singed and their function within the branching dendrite and thus provides an overview of how these actin modulatory proteins could work in concert within the process of dendritic branch formation [13].

Altogether, such progress will enable the identification of the variations in the programmes responsible for unique dendrite morphologies. Together, these findings will reveal how different actin building blocks are responsible for the diversity of dendrite morphologies that we have admired since the beginning of neuroscience research.
